# Unused Natural Variation Can Lift Yield Barriers in Plant Breeding

**DOI:** 10.1371/journal.pbio.0020245

**Published:** 2004-08-24

**Authors:** Amit Gur, Dani Zamir

**Affiliations:** **1**The Robert H. Smith Institute of Plant Sciences and Genetics in Agriculture, Faculty of AgricultureThe Hebrew University of Jerusalem, RehovotIsrael

## Abstract

Natural biodiversity is an underexploited sustainable resource that can enrich the genetic basis of cultivated plants with novel alleles that improve productivity and adaptation. We evaluated the progress in breeding for increased tomato *(Solanum lycopersicum)* yield using genotypes carrying a pyramid of three independent yield-promoting genomic regions introduced from the drought-tolerant green-fruited wild species *Solanum pennellii.* Yield of hybrids parented by the pyramided genotypes was more than 50% higher than that of a control market leader variety under both wet and dry field conditions that received 10% of the irrigation water. This demonstration of the breaking of agricultural yield barriers provides the rationale for implementing similar strategies for other agricultural organisms that are important for global food security.

## Abstract 2

Dramatic improvements in crop yield (in tomatoes, in this case) can be achieved by breeding selected genetic regions from wild species into domesticated varieties

## Introduction

Plant evolution under domestication has led to increased productivity, but at the same time it has narrowed the genetic basis of crop species (Ladizinsky 1998). A major objective in modern breeding is to return to the wild ancestors of crop plants and employ some of the diversity that was lost during domestication for the improvement of agricultural yields under optimal as well as stress field conditions (Bessey 1906; Tanksley and McCouch 1997; Lee 1998; Zamir 2001). Most of the genetic variation present in wild species has a negative effect on the adaptation of plants to agricultural environments; hence, the challenge is to identify and utilize the advantageous traits in a breeding program. DNA markers have facilitated quantitative trait loci (QTL) mapping studies in segregating populations, showing that certain genomic regions derived from wild germplasm have the potential to improve yield, e.g. for rice (Septiningshi et al. 2003), wheat (Huang et al. 2003), barley (Pillen et al. 2003), soybean (Concibido et al. 2003), chickpea (Singh and Ocampo 1997), tomato (Bernacchi et al. 1998), and pepper (Rao et al. 2003). In the above studies, and many others that are not cited, plants in the segregating populations generally contained a number of wild-species chromosome segments which masked the magnitude of some of the favorable effects that were clearly identified for certain introgressed alleles. As a result, the yield-promoting QTL did not have a substantial contribution to the phenotype and the best lines were inferior to intensively bred varieties that are in wide commercial cultivation. A major advantage for the above populations is that they can easily lead to the development of introgression lines that are discussed below. The main question addressed in the present study is whether it is possible to incorporate favorable wild-species QTL into genetic backgrounds that will consistently out-perform the leading varieties in the market.

**Figure 1:**
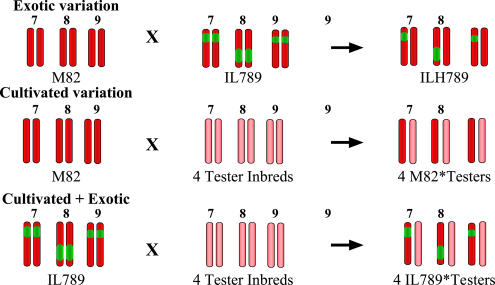
Genetic Sources of BY Variation (Exotic variation) Since M82, the multiple-introgression line (IL789), and their hybrid IL789 × M82 (ILH789) differ only in three S. pennellii segments (green chromosomes), any BY difference between them is associated with the exotic allelic variation. (Cultivated variation) Yield differences between M82 (red chromosomes), the four tomato tester inbreds (pink chromosomes), and their hybrids (M82 × Testers) result from allelic variation present in the cultivated tomato gene pool. (Cultivated + Exotic) The yield of the hybrids of IL789 with the four testers (IL789 × Testers) results from both cultivated and exotic variation.

To enhance the rate of progress of breeding based on wild-species resources, we developed a population of tomato segmental introgression lines (ILs). The ILs comprised marker-defined genomic regions taken from the drought-tolerant wild species Solanum pennellii and introduced (through genetic crosses) onto the genetic background of the elite inbred variety M82 (Eshed and Zamir 1995; see Knapp et al. 2004 for the new taxonomic classification of tomato species in the genus *Solanum*). The ILs constitute an “exotic library” where the entire wild-species genome was partitioned among 76 lines each carrying a single homozygous introgressed segment. Implementation of this resource for QTL mapping is based on the nearly isogenic nature of the lines such that any phenotypic difference between M82 and an IL, or the hybrid of M82 with an IL (ILH), is attributable to the S. pennellii genomic segments (Figure 1). Similar population structures were recently shown to greatly facilitate the detection of naturally occurring variation in inbred mice (Singer et al. 2004).

Phenotyping is the rate-limiting component in the utilization of such an exotic genetic resource. This is particularly true for quantitative traits where the reproducibility of the phenotype has to be evaluated in different seasons, environments, and genetic backgrounds. Over the past ten years the ILs and their hybrids have been assayed for yield-associated traits, and the data are presented, *in silico,* in a search engine that displays a range of statistical and graphical outputs that describe the components of the genetic variation (http://zamir.sgn.cornell.edu/; Gur et al. 2004). A review of the tomato QTL data and results of the QTL mapping studies from other species indicate that it is unlikely that a single introgression will induce a striking improvement in a yield-associated phenotype. However, pyramiding a number of independent introgressions in a single genotype, each with a positive effect on the desired trait, could be a strategy to greatly improve performance. The nearly isogenic nature of the ILs provides a relative advantage over other segregating populations in the rapid implementation of a pyramiding approach through crosses and marker analysis. We demonstrate here that the ILs make an efficient reagent for the discovery and utilization of genes that underlie traits of agricultural value and constitute a resource to explore the interactions among independent yield-associated QTL. We show that the pyramiding of independent yield-promoting segments can lead to novel varieties that reproducibly increase productivity relative to leading commercial genotypes both under normal cultivation conditions and in the stress environment of drought.

## Results/Discussion

### Exotic Variation

In “ketchup tomatoes,” which are used to produce various concentrated products, agricultural yield is made up of the total weight of the fruits harvested per unit area (yield [Y], measured in kg/m^2^) and their soluble-solids content (mainly the sugars glucose and fructose), which is measured in refractometer brix (B) units (expressed as a percentage). Therefore, agricultural yield of processing tomatoes is the total sugar output per unit area (brix × yield [BY], measured in g/m^2^); the industry is searching for varieties that excel in BY. Our experiments were conducted in wet and dry fields, where the BY of the control variety M82 in the dry conditions was only 50% of that produced in the wet treatment, which received 10-fold greater irrigation (184 g/m^2^ and 353 g/m^2^, respectively; average BY from 3 y).

**Figure 2:**
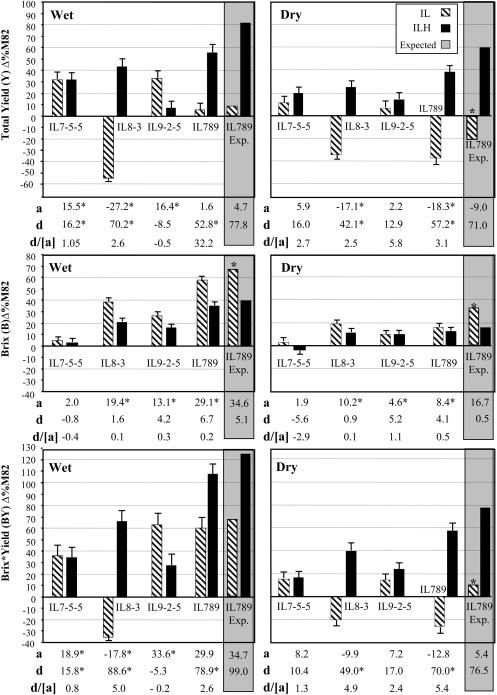
Pyramiding of S. pennellii Introgressions That Increase Agricultural Yield Components Introgression lines IL7-5-5, IL8-3, IL9-2-5, and IL789, which combines all three segments, were compared to M82 (percent difference from M82) in a homozygous (IL) and heterozygous (ILH) condition in wet and dry fields (1 plant/m^2^). The bars represent total yield (Y), brix (B), and brix × yield (BY) means (± standard error) from three growing seasons; these data were pooled, since no season × genotype interactions were found. The base line represents M82, where the mean BY values of M82 from the three seasons were 353 g/m^2^ in the irrigated treatment (455 g/m^2^ in 2001, 285 g/m^2^ in 2002, and 320 g/m^2^ in 2003) and 184 g/m^2^ in the dry treatment (244 g/m^2^ in 2001, 186 g/m^2^ in 2002, and 122 g/m^2^ in 2003). The additive effect *(a)* is half of the difference between each IL and M82. The dominance deviation *(d)* is the difference between ILH and the mid-value of its parents. Values marked by an asterisk are significant (*p* < 0.05). The bars in the gray background and their corresponding *a* and *d* values represent the expected values of IL789 and ILH789 assuming complete additivity of the introgression effects. Asterisks above the expected-value bars represent significant deviations from the observed means for IL789 or ILH789 as determined by a *t* test at a confidence level of 95%. All experiments were transplanted in a randomized block design with the following number of replications for each genotype under each irrigation regime: 2001, 10 replications; 2002, 15 replications; 2003, 15 replications.

In this study we focused on three independent introgressions from Chromosome 7 (IL7-5-5), Chromosome 8 (IL8-3), and Chromosome 9 (IL9-2-5) that affect the components of BY. Figure 2 summarizes the data of the yield components from three growing seasons in wet and dry fields. The data from the different years were pooled, as we detected no significant year *x* genotype interactions. IL7-5-5 was dominant in its effect on Y, as both the homozygous IL and heterozygous ILH increased Y in the wet fields by 30% compared to M82 and by 12% to 22% (nonsignificant) in the dry fields. IL7-5-5 did not affect B, while for BY it was dominant. IL8-3 was greatly inferior to M82 for Y (−55% and −34% for the wet and dry treatments, respectively), but the ILH increased Y by 45% and 25% (wet and dry, respectively). This result indicates a strong overdominant effect for the introgression (*d/[a]* = 2.5; see “Statistical analyses” for definitions). The homozygous IL had double the effect on B relative to the ILH; this resulted in a strong overdominant effect on BY in both environments (70% and 40% increases relative to M82 in the wet and dry fields, respectively; *d/[a]* = 5). The reduced Y of IL8-3 was caused by a pleiotropic effect of a leaf necrosis gene that was observed in all lines that were homozygous for this introgression; the necrosis was particularly severe in the dry treatment. IL9-2-5 significantly increased Y only in the homozygous condition in the wet treatment. For B and BY, ILH9-2-5 was intermediate between M82 and the homozygous IL, showing an additive mode of inheritance. The nature of the genes that improve BY in the above introgressions is unknown, with the exception of IL9-2-5, which harbors at least two QTL that affect the components of BY (Fridman et al. 2002). One of the QTL is *Brix9-2-5,* which resides in a 484-bp interval within the apoplastic invertase *(LIN5)* that increases sugar content of the fruit as a result of a modification of enzyme functions (Fridman et al. 2000 and unpublished data). *LIN5* and three other invertase family members reside on segmental duplications in the near-collinear genomes of tomato and potato. These chromosomal segments are syntenically duplicated in the model plant *Arabidopsis* and in rice, thus facilitating the research of synteny-based orthologs and their relationship to yield components (Fridman and Zamir 2003).

The three S. pennellii segments were pooled, using marker-assisted selection, into a single M82 line designated IL789 (homozygous for IL7-5-5, IL8-3, and IL9-2-5). IL789 showed a strong interaction with the environment: In the wet treatment it produced a Y similar to that produced by M82, while in the dry fields Y was reduced by 36% as a result of the pleiotropic effect of the recessive leaf necrosis gene on IL8-3. In the heterozygous condition IL789 dramatically increased Y in both field environments, and combined with the increases in B, ILH789 improved BY by 109% in the wet field and 58% in the dry fields. As described in earlier studies (Eshed and Zamir 1995) and demonstrated here for IL789, the positive effects of the wild introgressed segments on yield-associated traits were often more pronounced in the heterozygous condition due to linked deleterious recessive genes originating from S. pennellii.

In a previous study based on the ILs, Eshed and Zamir (1996) showed less-than-additive epistatic interactions for yield QTL. For a large number of lines carrying pairs of different introgressed segments, they detected a trend of lower BY values than were expected based on the sum of the effects of the individual ILs. This phenomenon was more pronounced for combinations of ILs that affected the same component of BY (either B or Y). The less-than-additive mode of interaction was suggested as an underlying genetic model to explain canalized characters, where the phenotype is kept within narrow boundaries despite genetic and environmental disturbances. For quantitative traits affected by a large number of QTL, the less-than-additive interaction ensures that a “loss” of an allele will have a minimal effect on the canalized phenotype. In the present study we compared the observed phenotypic values of IL789 and ILH789 with the expected value based on the assumption of additivity of the effects of the three introgressions that were pyramided into these lines. In the wet treatment all the observed values for IL789 were lower than expected; however, this trend was statistically significant only for B. In the dry fields the recessive leaf necrosis gene on IL8-3 exerted a strong epistatic effect which nullified the contribution of the other introgressions. Thus in all cases the observed values for IL789 were lower than expected and very similar to those for IL8-3 (Figure 2). From the plant breeding point of view, it is noteworthy that by pyramiding three heterozygous introgressions that affect the different components of BY, we achieved in ILH789 81% and 70% additivity (wet and dry, respectively) of the effects of the individual ILHs. Thus the heterozygous wild-species introgression pyramid improved BY in the genetic background of M82 by 109% in the wet fields and 58% in the dry fields.

### Cultivated Variation

**Figure 3:**
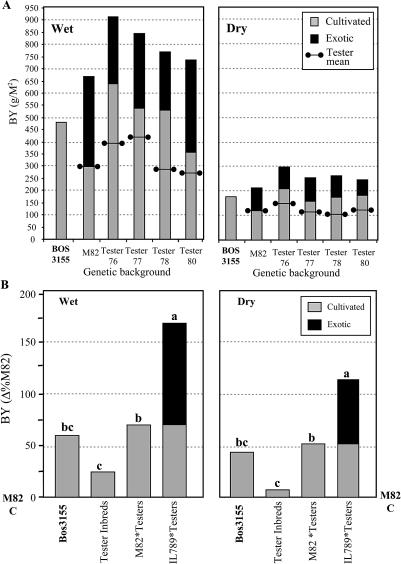
Contribution of Cultivated and Exotic Variation to BY (A) The contribution of cultivated and exotic variation to BY in five genetic backgrounds. BY phenotypes in the Akko wide-spacing wet and dry experiments that involved four independent tester inbreds and their hybrids with M82 and IL789 are shown. Included are mean values for a control background of M82 and BOS3155. The four inbreds are represented as horizontal lines with circles in the gray bars. Experiments were transplanted in a randomized block design with 20 replications for each genotype under each irrigation regime. In all cases BY of the hybrids containing the exotic introgressions (IL789 × Testers) was significantly higher than that of their nearly isogenic cultivated tomato hybrids (M82 × Testers; *t* test, *p* < 0.01). The exotic effect, which represents the BY differences between the IL789 × Testers hybrids and the M82 × Testers hybrids, was consistent for all genetic backgrounds in each of the irrigation regimes. This was determined by genetic background × exotic effect two-way ANOVA (*p* for the interaction is 0.75 for the wet treatment and 0.88 for the dry field). (B) The mean contribution of cultivated and exotic variation to BY in wet and dry fields. BOS3155 is a leading commercial tomato hybrid that was used as a reference. Values of tester inbreds, M82 × Testers, and IL789 × Testers (shown as Δ% from M82) represent the means of the four genotypes included in each group (see Figure 3A). Base line and the letters attached to it represent M82. Means for each irrigation treatment with different letters are significantly different using a multiple-range means comparison (Tukey-Kramer; *p* < 0.01). The deduced exotic effect on BY is marked as black bars, and the contribution of the cultivated variation to BY is marked in gray. The absolute BY values of M82 were 303 g/m^2^ in the wet treatment and 122 g/m^2^ in the dry treatment.

Our breeding program within the Solanum lycopersicum gene pool over the past several years has generated tester inbreds of different origins that give a sampling of the genetic diversity of processing tomatoes. Four testers, whose hybrids with M82 exhibited the highest BY, were selected for this experiment, which was aimed at exploring the breeding potential of the genetic variation within the processing tomato germplasm (Figure 3A). The BY values of the inbred testers in the wet and dry treatments were not significantly different from that of M82, whereas the four hybrid combinations with M82 had higher mean BY in both environments (71% in the wet treatment and 51% in the dry treatment; Figure 3B). This improvement over the parents reflects the genetic variation present in the cultivated tomato gene pool, which was expressed as hybrid vigour originating from crossing of the preselected diverse inbreds (see Figure 1). As a reference outgroup for the entire experiment we selected the commerical hybrid BOS3155, which has been a processing tomato market leader in California for the past five years (http://www.ptab.org/). BY of BOS3155 was in a range similar to that of our experimental hybrids, indicating that the experiment was conducted in elite genetic backgrounds.

### Combining Exotic and Cultivated Variation

A correct assessment of the potential of exotic QTL is in the context of high-yield genetic backgrounds—those close to the “yield barrier.” This was achieved by crossing IL789 with the four inbred tester lines in a manner that combined the contribution of both the cultivated and the exotic variation (see Figure 1). These IL789 × Testers hybrids were nearly isogenic to the M82 × Testers hybrids, and thus the differences between them reflect the effect of the exotic alleles on BY. The effects of the three heterozygous introgressions on BY were consistent in all the hybrid combinations, and no genetic background × exotic effect interactions were found in any of the environments (Figure 3A). The mean BY increase of the four IL789 × Testers hybrids, compared to M82, was 170% (wet) and 115% (dry), while the mean contribution of the M82 × Testers hybrids was 71% (wet) and 51% (dry) (Figure 3B). Based on the consistent effect of the introgressions in the different genetic backgrounds, we could estimate the contribution of the S. pennellii pyramid as the mean difference between the nearly isogenic hybrid groups: 100% (wet) and 65% (dry). These estimates for the exotic effects on BY are very close to those obtained in the uniform M82 background, indicating additivity of the exotic and cultivated effects (see Figure 2).

**Figure 4:**
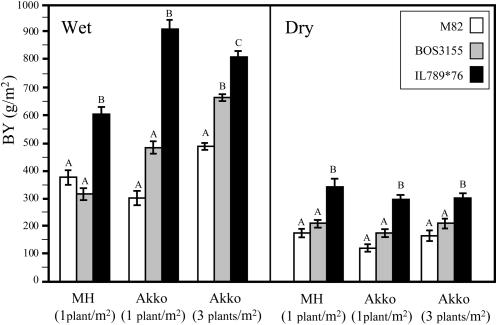
BY Phenotypes of M82, BOS3155, and the Best Hybrid Combination (IL789 × 76) in Six Independent Trials Plants were grown in two locations, under two planting densities and two irrigation regimes. The locations were (i) the Western Galilee Experimental Station in Akko and (ii) Kibbutz Mevo-Hama (MH) in the Golan Heights. The planting densities were (i) single plants (SP; 1 plant per m^2^) and (ii) plots (14 plants per 4 m^2^, or 3.5 plants/m^2^). The irrigation regimes were (i) wet (320 m^3^ of water per 1,000 m^2^ of field throughout the growing season) and (ii) dry (30 m^3^ of water per 1,000 m^2^ of field). All experiments were transplanted in a randomized block design with the following number of replications: Akko-SP-wet, 15 replications; Akko-SP-dry, 20 replications; Akko-plots-wet, 8 replications; Akko-plots-dry, 8 replications; MH-SP-wet, 15 replications; MH-SP-dry, 15 replications. Means not connected by the same letter are significantly different using a multiple-range means comparison (Tukey-Kramer; *p* < 0.01).

The highest BY was measured for the hybrid of IL789 with inbred #76. This hybrid was compared with M82 and BOS3155 in six independent experiments that differed in location, planting density, and irrigation regime (Figure 4). The BY advantage of the IL789 × 76 hybrid relative to M82 was not accompanied by other negative traits originating from the wild species and was observed in all field environments, ranging from 60% in Mevo-Hama with wide spacing and wet treatment to 200% in Akko under similar conditions. Significantly, the mean BY improvement of this IL789 × 76 hybrid over the market leader BOS3155 was 67% in the irrigated conditions and 58% in the dry conditions.

The results of IL789 × 76 and the control varieties in the different environments provided the means to explore genotype × environment (G × E) interactions and the stability of BY improvement associated with the heterozygous S. pennellii introgressions (Table 1). Generally, strong G × E interactions of new varieties indicate the lack of a predictable response, which is undesirable in breeding (Dudley and Moll 1969). In the wet fields we detected significant genotypic, environmental, and G × E interaction effects for the varieties tested. However, the IL789 hybrid always had significantly higher BY than the commercial varieties, and the interaction was caused in part by differences between the commercial varieties M82 and BOS3155 (Figure 4). In the dry trials there was a highly significant genotypic effect and marginally significant effects for the environment; no interaction between the two components was detected, and IL789 × 76 had higher BY in all experiments. This analysis highlights the potential of wild germplasm to affect yield stability in diverse environments, which has long been recognized as an important objective in plant breeding.

**Table 1:**
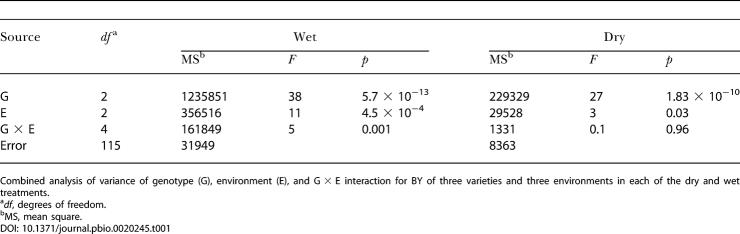
ANOVA of Genotype by Environment Interaction for BY in IL789 × 76, BOS3155, and M82

Combined analysis of variance of genotype (G), environment (E), and G × E interaction for BY of three varieties and three environments in each of the dry and wet treatments

^a^ *df*, degrees of freedom

^b^MS, mean square

We have demonstrated that an IL population derived from a wild tomato species, with no yield potential, can make a wide array of previously unexplored genetic variation rapidly available to plant breeders to improve crop productivity. The effectiveness of the introgressions in diverse genetic backgrounds indicates that alleles similar to those of the wild species are not present in the cultivated tomato gene pool. The results presented here using the tomato ILs establish a genetic infrastructure to explore the molecular basis underlying yield heterosis. With the coming sequence of the tomato genome it will be easier to isolate those factors that are responsible for the strong overdominant effects, such as those observed for IL8-3 and some additional lines that are described in the IL phenotypic database Real Time QTL (Gur et al. 2004). Finally, our approach of pyramiding beneficial wild-species chromosome segments provides an alternative to the genetically modified organism strategy for crop improvement and offers a new paradigm to revitalize plant breeding (Tanksley and McCouch 1997; Zamir 2001; Morgante and Salamini 2003; Koornneef et al. 2004). Can these results be extrapolated to the breeding of other crop plants? Wild species that are distantly related to crop plants can be viewed as vast naturally mutagenized resources where every gene and regulatory element has been refined and defined by evolution. We propose that for crops that rely on a rather narrow genetic basis (rice, wheat, soybean, etc.) and have rich biodiversity resources, the construction and screening of ILs will lead to dramatic improvements in yield and other quality traits that are important for human well-being (Rosegrant and Cline 2003). As we are able to make a wider range of natural genetic diversity accessible to breeders, we will make progress in improving our global food security.

## Materials and Methods

### 

#### Field trials

The results presented are from three growing seasons. In 2001 and 2002 all field trials were conducted at the Western Galilee Experimental Station in Akko, Israel, at a wide-spacing planting density of 1 plant per m^2^. In 2003, trials were conducted in two locations: Akko and Mevo-Hama, in the Golan Heights. In Akko, experiments were both at wide-spacing planting density and in plots of 14 plants per 4 m^2^ (3.5 plants/m^2^). In Mevo-Hama all experiments were at the wide-spacing density. The seedlings were grown in a greenhouse for 35–40 d and then transplanted in the field, at the beginning of April in Akko, and at the beginning of May in Mevo-Hama. In all seasons and locations both wet and dry trials were conducted. Both the wet and dry fields started the growing season at “field capacity,” which represents the maximum amount of water that the soil could hold. For the dry treatment only 30 m^3^ of water was applied per 1,000 m^2^ of field immediately after transplanting. In the wet treatment 320 m^3^ of water was applied per 1,000 m^2^ of field throughout the growing season according to the irrigation protocols in the area. All experiments were transplanted in a randomized block design.

#### Genotyping and phenotyping

To ensure the nearly isogenic nature of the ILs, we bred an M82 line that was heterozygous for all three introgressions and used RFLP markers to genotype 128 F2 plants segregating for the three S. pennellii genomic segments (CT252 for IL7-5-5, CT148 for IL8-3, and GP263 for IL9-2-5). Lines homozygous for each of the segments and IL789 homozygous for all three introgressions were selected and verified with RFLP markers that flanked the introgressed segments from both ends (http://www.sgn.cornell.edu/). Phenotyping of the plants for Y, B, and BY was performed according to published protocols (Fridman et al. 2002).

#### Statistical analyses

Statistical analyses were performed using the JMP V.5 software package (SAS Institute, Cary, North Carolina, United States). Mean values for the parameters measured for the tested genotypes were compared using the “Fit Y by X” function and “Compare all pairs” (Tukey-Kramer). All calculations were performed with the phenotypic values, while some of the results are presented as the percent difference from M82. The additive effect *(a)* was half of the difference between each IL and M82, and its significance level was determined by the comparison between the IL and M82. The dominance deviation *(d)* is the difference between ILH and the mid-value of its parents. Its significance level was calculated by contrasting the ILH (+1) with M82 (−0.5) and the appropriate IL (−0.5). The degree of dominance for each introgression *(d/[a])* was calculated by dividing the mean dominance deviation by the mean additive effect. Deviation of the observed yield component values of the pyramided genotypes (IL789 and ILH789) from the expected values based on the assumption of additivity of the effects of the individual introgressions was tested using a *t* test at *p* < 0.05. G × E interaction was tested using a two-way ANOVA.

## Abbreviations

B: brix
BY: brix × yield
G × E: genotype × environment
IL: introgression line
ILH: IL hybrid
QTL: quantitative trait loci
Y: yield
